# Morphologic Features of Myopic Choroidal Neovascularization in Pathologic Myopia on Swept-Source Optical Coherence Tomography

**DOI:** 10.3389/fmed.2020.615902

**Published:** 2020-12-23

**Authors:** Jiamin Xie, Qiuying Chen, Jiayi Yu, Hao Zhou, Jiangnan He, Weijun Wang, Ying Fan, Xun Xu

**Affiliations:** ^1^Department of Ophthalmology, Shanghai General Hospital, Shanghai Jiao Tong University School of Medicine, Shanghai, China; ^2^National Clinical Research Center for Eye Diseases, Shanghai, China; ^3^Shanghai Key Laboratory of Ocular Fundus Disease, Shanghai, China; ^4^Shanghai Engineering Center for Visual Science and Photo Medicine, Shanghai, China; ^5^Shanghai Engineering Center for Precise Diagnosis and Treatment of Eye Diseases, Shanghai, China; ^6^Department of Preventative Ophthalmology, Shanghai Eye Disease Prevention and Treatment Center, Shanghai Eye Hospital, Shanghai, China

**Keywords:** choroidal thickness, myopic choroidal neovascularization, scleral thickness, scleral perforating vessels, pathologic myopia

## Abstract

**Purpose:** To investigate the morphologic features and identify the risk factors of myopic choroidal neovascularization (CNV).

**Methods:** Eighty-eight eyes of 69 consecutive patients with myopic CNV were included in this study. About 109 eyes of 78 pathologic myopia patients without myopic CNV were randomly selected as the control group. Morphologic features and parameters including scleral thickness (ST), choroidal thickness (CT), posterior staphyloma height and the presence of scleral perforating vessels were obtained and measured by swept-source optical coherence tomography (SS-OCT). Binary logistic regression analysis was performed to identify the risk factors for myopic CNV.

**Results:** Patients with myopic CNV had relatively shorter axial length (*P* < 0.001) and thicker sclera (*P* < 0.001) compared to those without. After adjusting age, gender and axial length, thick sclera (OR = 1.333, *P* < 0.001 per 10-μm increase) and thin choroid (OR = 0.509, *P* < 0.001 per 10-μm increase) were associated with the presence of myopic CNV. Scleral perforating vessels were detected in the area of myopic CNV in 78.67% of the subjects.

**Conclusions:** A relatively thicker sclera and a thinner choroid are the biological indicators for myopic CNV on SS-OCT. Scleral perforating vessels may also play a pivotal role in the formation of myopic CNV.

## Introduction

Myopic choroidal neovascularization (CNV) is a common vision-threatening complication in pathologic myopia (PM) (1). The prevalence has been estimated to be 5.2 to 11.3% among individuals with PM (2). The long-term visual prognosis of myopic CNV is extremely poor without treatment. It has been reported that the visual acuity of myopic CNV deteriorated to 20/200 or worse in ~89 and 96% of eyes in 5 years and 10 years, respectively (3). Intravitreal anti-VEGF therapy is the standard-of-care and first-line treatment for myopic CNV (4). Several studies have confirmed that early diagnosis and treatment of myopic CNV predicts a better visual outcome (5, 6). However, it is often difficult for patients with PM to notice new occurrences of myopic CNV because of the already impaired vision caused by other pathologies, which will finally lead to irreversible vision loss (4).

Optical coherence tomography (OCT) is a non-invasive imaging tool that has been used to diagnose and monitor treatment response in myopic CNV (7). Longer wavelength (1.050–1.060 nm) and deep penetrance swept-source OCT (SS-OCT) demonstrates its superiority in myopic eyes as it can provide clear visualization of the sclera and orbital fat tissue in myopes with longer axial lengths (7). In OCT image, myopic CNV presents as a highly reflective area contiguous above the RPE (type 2 CNV), usually with minimal subretinal fluid (4). Three different phases of myopic CNV have been identified based on the characteristics observed on OCT: active, scar, and atrophic phase (7).

Until recently, only few studies have described the risk factors associated with myopic CNV on OCT, and they mainly focus on choroidal morphology (8, 9). Sclera plays a pivotal role in determining eye size and the development of myopia (10). Despite its importance, the scleral morphology associated with the complications of myopia has not fully been explored yet. As such, we intend to supplement the current understanding of scleral morphology in myopic CNV.

Therefore, we aim to investigate the morphologic features and to identify the risk factors of myopic CNV on SS-OCT. Furthermore, it may help to find the high-risk groups among large-scale populations in future investigations.

## Methods

### Participants

The study included 88 eyes of 69 consecutive patients with myopic CNV who had visited high myopia clinic in ophthalmological department of Shanghai General Hospital from July 2017 to July 2019. A myopic CNV was defined as a CNV that was present in eyes with PM (11). PM was defined as eyes having myopic maculopathy equal to or more severe than diffuse atrophy (12, 13). A cohort of 109 eyes of 78 PM participants were randomly selected to serve as the control group. The inclusion criteria were as follows: a SER < −6 diopters (D) or an AL ≥ 26 mm with PM; normal anterior chamber angles; normal optic nerve head (ONH) without glaucomatous changes; and no other ocular diseases. Exclusion criteria were as follows: previous intraocular or refractive surgery other than cataract surgery; features suggesting that the CNV may be associated with AMD, multifocal choroiditis or angioid streaks; Poor-quality images were also excluded. The diagnosis of a myopic CNV was based on the presence of a highly reflective area contiguous above the RPE (type 2 CNV), usually with minimal subretinal fluid (4, 14). Considering that myopic CNV is often bilateral (15), the fellow eye of the pre-existing myopic CNV patients were not included in the control group. This study was conducted in accordance with the Declaration of Helsinki and was approved by the ethics committee of Shanghai General Hospital. Informed consent was obtained from each patient.

### Examinations

All study participants underwent a comprehensive ophthalmic examination, including measurements of slit-lamp biomicroscopy, intraocular pressure (IOP, Full Auto Tonometer TX-F; Topcon, Japan), best-corrected visual acuity (BCVA), axial length (IOL Master; Carl Zeiss, Tubingen, Germany) and assessment of the refractive error using an autorefractor instrument (model KR-8900; Topcon, Japan). The BCVA was converted into the logarithm of minimal angle resolution (logMAR). FFA was obtained by Heidelberg Spectralis HRA (Heidelberg Engineering, Heidelberg, Germany). Swept-source optical coherence tomography (SS-OCT, model DRI OCT-1 Atlantis; Topcon, Japan) was used to capture color fundus photographs and to measure the scleral thickness (ST) and choroidal thickness (CT). The OCT scanning protocols included a length of 9 mm with 12 equal radial meridian scans centered on the fovea.

### Classification and Definition

According to the International Photographic Classification and Grading System, myopic maculopathy (MM) was classified based on fundus photographs into tessellated fundus (C1), diffuse chorioretinal atrophy (C2), patchy chorioretinal atrophy (C3), and macular atrophy (C4) (12). Scleral perforating vessels were defined as uniform hypo-reflective structures within the scleral stroma and macular area on SS-OCT B-scanned images (Figures 2B,F) (16). Dome-shaped macula (DSM) was characterized as an inward bulge of the retinal pigment epithelium of more than 50 μm in the vertical and/or horizontal direction on OCT examination (17). Myopic CNV was classified into three phases on OCT (Figure 2): the active phase (a hyperreflective elevation with or without exudation or hemorrhage. All features, including subretinal fluid, subretinal hyperreflective exudation and the fuzzy borders of CNV and a lack of RPE coverage indicate an active phase of a CNV), the scar phase (a hyperreflectivity in the inner surface of the CNV, with attenuation of the tissue bellow, which is also known as “Fuch's spot”), and the atrophic phase (also known as CNV-related macular atrophy with enhanced choroidal reflectivity corresponding to the area of chorioretinal atrophy, and is characterized by the formation of a hole in Bruch's membrane) (4, 7, 18). Fundus fluorescein angiography (FFA) was performed when needed. Two masked ophthalmologists (J.X. and Q.C.) performed the classification of myopic CNV. Discrepancies were adjudicated by a senior retinal specialist (Y.F.).

### Measurements

**Figure 1:**
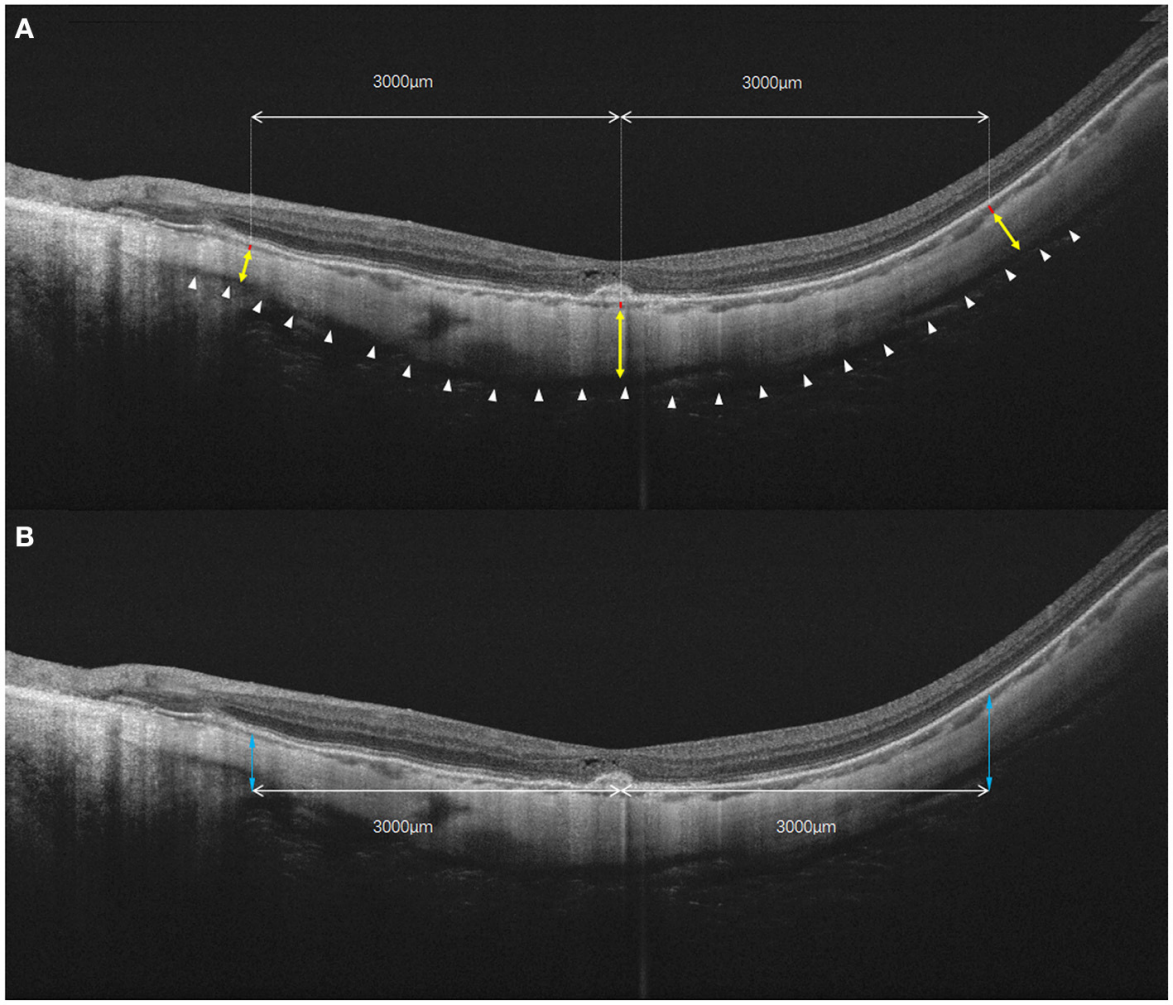
The diagrammatic sketch for the measurements of choroidal and scleral thickness, as well as the height of posterior staphyloma. **(A)** Choroidal and scleral thickness was measured along the perpendicular axis to the curvature of the retinal pigment epithelium. Yellow double arrow: scleral thickness measured in fovea and 3 mm nasal and temporal to the fovea. Red bar: choroidal thickness measured in fovea and 3 mm nasal and temporal to the foveal. White arrowheads: outer surface of the sclera. **(B)** The height of the posterior staphyloma (double blue arrow) was measured as the vertical distance from the subfoveal retinal pigment epithelium line to the point at 3 mm from both sides of the fovea. In the vertical scan, choroidal and scleral thicknesses also were measured at 3 mm superior and inferior to the fovea.

Two trained graders (J.Y. and J.H.) blinded to the study independently measured the thickness of each layer at the fovea, 1.5 mm and 3.0 mm from the macula on horizontal and vertical radial OCT scan lines. The CT was defined as the vertical distance between the outer border of the RPE and the choroidal–scleral interface. The ST was defined as the vertical distance between the choroidal–scleral interface and the outer scleral border. If the absolute difference between two measurements was >20 μm for the sclera or 10 μm for the choroid, the measurements were repeated until the absolute difference was within the set limits (19). The height of the posterior staphyloma (PS) was the vertical distance from the subfoveal retinal pigment epithelium line to 3 mm nasal, temporal, superior, and inferior to the fovea (8). The relative height of the PS was expressed as either a positive or a negative number depending on the edge located anteriorly or posteriorly. The absolute height of PS is used in some specific analyses. After adjusting the magnification of the AL, the ST, CT, and PS height on the OCT B-scan were measured by the built-in software (20). The measurements from the two graders were averaged. The 1.5 mm or 3.0mm average to the fovea defined as the average measurements of 1.5 mm or 3.0 mm superior, inferior, temporal, and nasal to the fovea center. The method used to measure CT, ST, and PS height is shown in Figure 1.

### Statistical Analysis

Statistical evaluation was performed using SPSS software (IBM SPSS Statistics 21; SPSS, Inc, Chicago, IL). Categorical variables, rank variables, and continuous variables were analyzed with the Chi-square test, Wilcoxon test and Mann-Whitey *U* test, respectively. Kruskal-Wallis test was used for comparisons of myopic CNV subtypes. Spearman's correlation was used to identify the correlations among ST/CT and AL. Binary logistic regressions were performed to evaluate the independent associations of myopic CNV occurrence, with adjustment for age, gender, and AL. Generalized estimating equations were used to account for correlation between left and right eyes of the same patient. All data were expressed as mean ± standard deviation or proportions as appropriate. A *P-*value < 0.05 was considered statistically significant.

## Results

The generalized estimating equation regression models revealed that there were no significant differences in ocular biometry between the two eyes; thus, it was unnecessary to adjust for associations between the two eyes.

Eighty-eight eyes of 69 patients diagnosed as myopic CNV and 109 eyes of 78 patients served as control group, were included in this study. Mean age of patients was 57.09 ± 11.93 years. Forty-one (27.9%) were men and 106 (72.1%) were women. Among eyes with myopic CNV, 41 eyes of 33 patients had received anti-VEGF treatment before the recruitment. None of them had been treated with photodynamic therapy. CNV was subfoveal in 68 (77.27%) eyes and juxtafoveal in 20 (22.73%) eyes. The mean CNV lesion size is 0.66 ± 0.77mm^2^. Patient demographic data is shown in Table 1. Patients with myopic CNV showed less myopic refractive error (−13.36 ± 4.52 vs. −16.05 ± 3.76 D, *P* < 0.0001, after excluding pseudophakic eyes), shorter axial length (29.51 ± 1.42 vs. 30.51 ± 1.78 mm, *P* < 0.0001) and worse BCVA (0.83 ± 0.69 vs. 0.39 ± 0.26, *P* < 0.0001) than those without myopic CNV.

**Table 1:** Comparisons of clinical characteristics between eyes with and without myopic CNV in PM.

**Characteristics**	**Eyes with myopic CNV**	**Eyes without myopic CNV**	***P***
Number of eyes (No. of persons)	88 (69)	109 (78)	
Age, y	58.88 ± 12.22	55.86 ±11.66	0.087
Gender, Male/Female	22/66	36/73	0.268
Eye, Right/Left	43/45	54/55	0.925
Axial length, mm	29.51 ± 1.42	30.51 ± 1.78	<0.001^*^
BCVA, logMAR	0.83 ± 0.69	0.39 ± 0.26	<0.001^*^
DSM (%)	13 (14.77%)	9(8.26%)	0.149

*CNV, choroidal neovascularization; PM, pathologic myopia; BCVA, best-corrected visual acuity; logMAR, logarithm of minimum angle of resolution; DSM, dome-shaped macula*.

* *Significant difference*.

**Figure 2:**
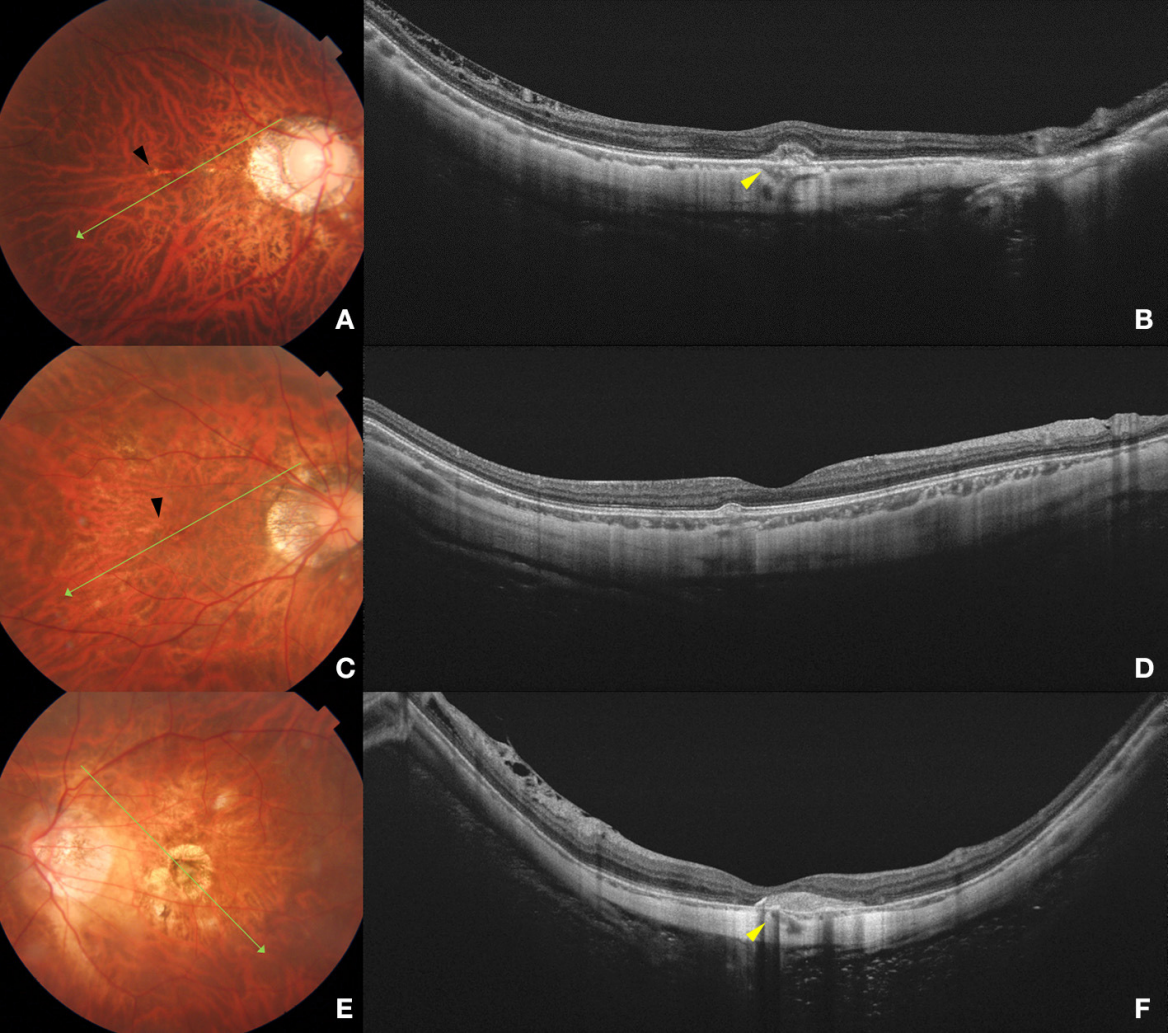
Fundus photograph and swept-source optical coherence tomography (SS-OCT) images of different phases of myopic CNV. Left column **(A,C,E)** shows color fundus photographs. The long green arrow shows the scanned line examined by SS-OCT. Right column **(B, D, F)** shows the corresponding SS-OCT image. **(A)** A 32-year-old young male with peripapillary diffuse atrophy and an active CNV. Mild bleeding and lacquer crack (black arrow) are observed in the macular area. **(B)** B-scan SS-OCT shows a type 2 CNV with minimal subretinal hyperreflective exudation. Scleral perforating vessel (yellow arrowhead) penetrated around CNV. The subfoveal scleral thickness is 288 μm. **(C)** A 40-year-old female with peripapillary diffuse choroidal atrophy and a scar phase myopic CNV. Lacquer cracks (black arrow) are shown in the macular area. **(D)** SS-OCT shows scarred CNV as a small RPE elevation with intact ellipsoid zone and ELM. The subfoveal scleral thickness is 360 μm. **(E)** A 61-year-old female with CNV-related macular atrophy. Macular atrophy around the myopic CNV is observed in the macular area. **(F)** SS-OCT shows a scarred CNV with a scleral perforating vessel (yellow arrowhead) penetrating below the CNV lesion. The subfoveal scleral thickness is 220 μm.

**Figure 3:**
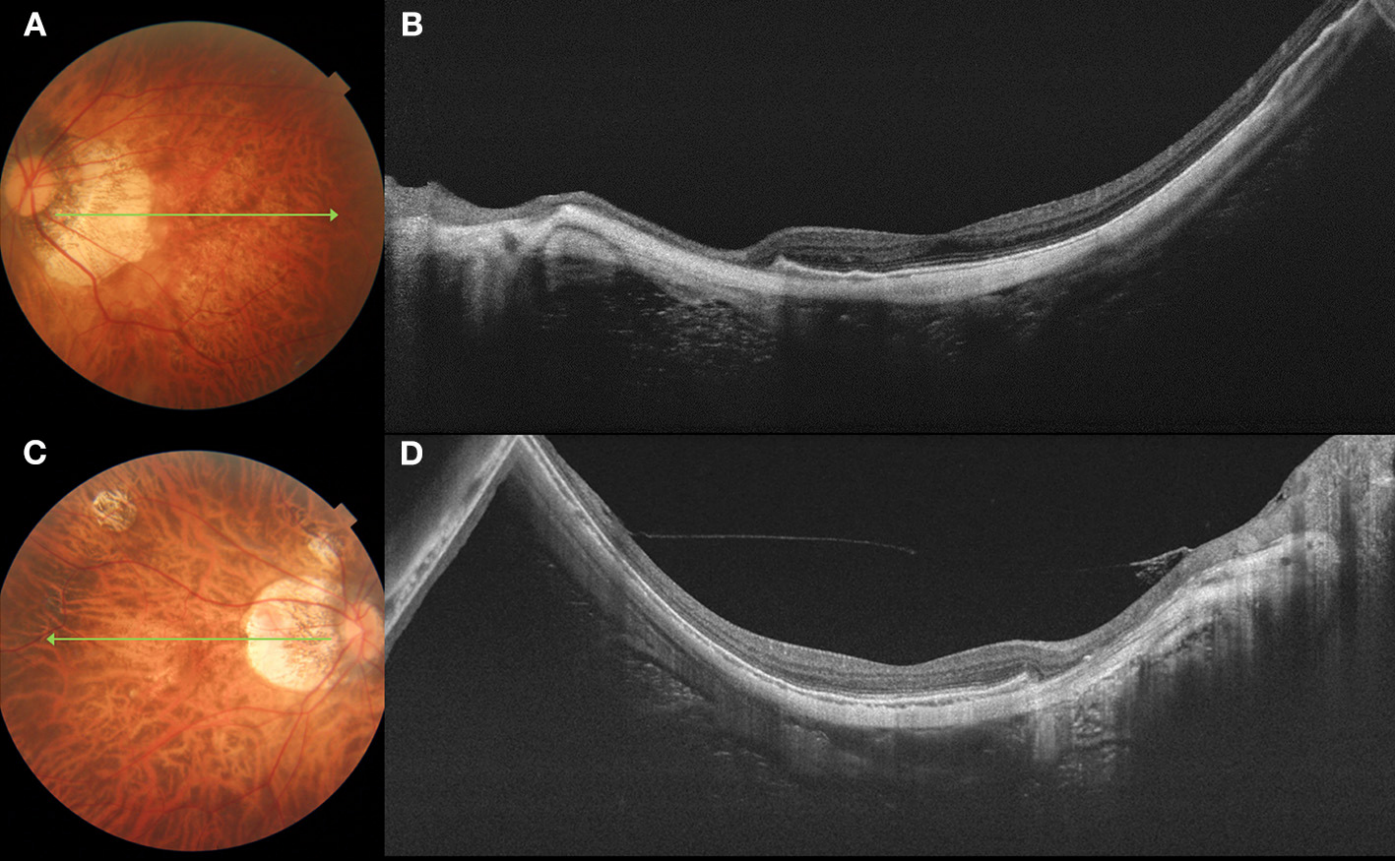
Fundus photograph and swept-source optical coherence tomography (SS-OCT) images of pathologic myopia eyes with no myopic CNV. Left column **(A,C)** shows color fundus photographs. The long green arrow shows the scanned line examined by SS-OCT. Right column **(B,D)** shows the corresponding SS-OCT image. **(A)** A 62-year-old female with diffuse atrophy fundus. **(B)** SS-OCT shows a relative thin sclera. The subfoveal scleral thickness is 207 μm. **(C)** A 46-year-old female with patchy atrophy. **(D)** SS-OCT shows a relative thin sclera, with subfoveal scleral thickness is 135 μm.

After exclusion of eyes with DSM (*n* = 22), 75 eyes with myopic CNV (Figure 2) were compared to 100 eyes without myopic CNV (Figure 3) on SS-OCT (Table 2). In eyes with myopic CNV, ST was significantly thicker at subfoveal region (290.89 ± 84.51 vs. 226.24 ± 58.38 μm, *P* < 0.001), 1.5 mm (234.76 ± 63.03 vs. 182.79 ± 44.99 μm, *P* < 0.001) and 3.0 mm (212.24 ± 51.28 vs. 172.61 ± 40.45 μm, *P* < 0.001) average to the fovea. The average ST/CT was also significantly larger in myopic CNV (12.74 ± 6.12 vs. 10.61 ± 4.70, *P* = 0.046). However, CT, average relative and absolute PS height showed no significant difference between two group.

**Table 2:** The comparison of parameters on SS-OCT between eyes with and without myopic CNV.

	**Eyes with myopic CNV (*n* = 75)**	**Eyes without myopic CNV (*n* = 100)**	***P***	***P'***
**ST**, **μm**				
Subfoveal	290.98 ± 84.51	226.24 ± 58.38	<0.001^*^	<0.001^*^
1.5 mm average	234.76 ± 63.03	182.79 ± 44.99	<0.001^*^	<0.001^*^
3.0 mm average	212.24 ± 51.28	172.61 ± 40.45	<0.001^*^	<0.001^*^
Total average	231.00 ± 57.52	182.83 ± 41.51	<0.001^*^	<0.001^*^
**CT**, **μm (67 eyes available in eyes with myopic CNV)**				
Subfoveal	16.84 ± 9.06	17.40 ± 12.14	0.842	0.669
1.5 mm average	21.79 ± 11.16	21.57 ± 15.70	0.231	0.769
3.0 mm average	23.23 ± 110.94	22.79 ± 17.88	0.139	0.964
Total average	21.83 ± 9.69	21.63 ± 15.23	0.156	0.916
Average ST/CT	12.74 ± 6.12	10.61 ± 4.70	0.046^*^	0.022^*^
Average relative PS height, μm	425.16 ± 162.43	483.48 ± 180.846	0.107	0.195
Average absolute PS height, μm	496.07 ± 134.90	520.89 ± 154.28	0.542	0.362

*SS-OCT, swept-source optical coherence tomography; CNV, choroidal neovascularization; ST, scleral thickness; CT, choroidal thickness; PS, posterior staphyloma; P', comparisons after adjusted age and gender*.

* *Significant difference*.

In eyes with diffuse atrophy (C2), the ones with myopic CNV showed relatively shorter axial length (*P* < 0.001) and thicker sclera at subfoveal (312.71 ± 93.79 vs. 234.62 ± 56.18 mm, *P* = 0.003), 1.5 mm (246.81 ± 71.24 vs. 190.56 ± 44.56 mm, *P* < 0.001) and 3.0 mm (224.98 ± 52.76 vs. 179.48 ± 41.33 mm, *P* < 0.001) to the fovea. However, CT showed no significant difference between eyes with and without myopic CNV (Table 3). In eyes with patchy atrophy (C3), those with myopic CNV showed relatively shorter axial length (*P* < 0.001), worse BCVA (*P* = 0.018) and thicker sclera at subfoveal (254.67 ± 52.95 vs. 204.96 ± 59.52 mm, *P* < 0.001), 1.5 mm (207.03 ± 42.55 vs. 163.09 ± 40.54 mm, *P* < 0.001) and 3.0 mm (197.83 ± 41.42 vs. 155.16 ± 32.76, *P* < 0.001) average to the fovea. However, the CT of eyes with myopic CNV was significantly thicker at 1.5 mm (*P* = 0.002) and 3.0 mm (*P* = 0.006) average to the fovea (Table 3).

**Table 3:** The comparison of characteristics and parameters on SS-OCT eyes with and without myopic CNV in C2 and C3.

	**Eyes with myopic CNV**		**Eyes without myopic CNV**		***P^***a***^***	***P^***b***^***	***P^***c***^***	***P^***d***^***
	**C2**	**C3**	**C2**	**C3**				
Number of eyes	32	26	73	27				
Age, y	59.03 ± 13.54	53.38 ± 13.25	57.33 ± 10.98	55.46 ± 10.40	0.227	0.936		
Axial length, mm	29.19 ± 1.56	29.84 ± 1.43	30.00 ± 1.59	31.76 ± 1.48	0.017^*^	<0.001^*^	0.022^*^	0.001^*^
BCVA, logMAR	0.54 ± 0.36	0.55 ± 0.28	0.40 ± 0.26	0.35 ± 0.22	0.050	0.018^*^	0.008^*^	0.025^*^
**ST,μm**								
Subfoveal	312.71 ± 93.79	254.67 ± 52.95	234.62 ± 56.18	204.96 ± 59.53	<0.001^*^	<0.001^*^	<0.001^*^	0.002^*^
1.5mm average	246.81 ± 71.24	207.03 ± 42.55	190.56 ± 44.56	163.09 ± 40.54	<0.001^*^	<0.001^*^	<0.001^*^	0.001^*^
3.0mm average	224.98 ± 52.76	197.83 ± 41.42	179.48 ± 41.33	155.16 ± 32.76	<0.001^*^	<0.001^*^	<0.001^*^	<0.001^*^
Total average	244.43 ± 63.61	208.23 ± 40.14	190.16 ± 41.55	164.22 ± 35.82	<0.001^*^	<0.001^*^	<0.001^*^	<0.001^*^
**CT,μm**								
Subfoveal	16.84 ± 9.61	17.22 ± 7.80	18.50 ± 12.92	14.62 ± 9.54	0.834	0.069	0.645	0.220
1.5mm average	23.83 ± 11.98	22.49 ± 9.27	24.53 ± 17.08	14.04 ± 7.52	0.582	0.002^*^	0.900	0.007^*^
3.0mm average	25.32 ± 11.74	20.83 ± 9.68	26.19 ± 19.70	14.15 ± 6.81	0.511	0.006^*^	0.806	0.001^*^
Total average	23.65 ± 10.80	21.11 ± 8.09	24.58 ± 16.72	14.13 ± 5.97	0.669	0.002^*^	0.894	0.005^*^
Average relative PS height, μm	389.56 ± 152.47	501.24 ± 178.94	434.21 ± 155.45	432.05 ± 131.17	0.058	0.570	0.056	0.528
Average absolute PS height, μm	482.82 ± 137.51	549.96 ± 136.66	483.19 ± 122.90	489.95 ± 95.71	0.326	0.442	0.169	0.300

*SS-OCT, swept-source optical coherence tomography; CNV, choroidal neovascularization; BCVA, best-corrected visual acuity; logMAR, logarithm of minimum angle of resolution; ST, scleral thickness; CT, choroidal thickness; PS, posterior staphyloma; P^a^, comparison between eyes with and without myopic CNV in C2; P^b^, comparison between eyes with and without myopic CNV in C3; P^c^, comparison between eyes with and without myopic CNV in C2 after adjusted age and gender; P^d^, comparison between eyes with and without myopic CNV in C3 after adjusted age and gender*.

* *Significant difference*.

In the subgroup of myopic CNV (Table 4), CNV-related macular atrophy showed a worse BCVA than active and scar CNV (*P* < 0.001). Active CNV had relatively thicker CT than CNV scar and CNV-related macular atrophy group at subfoveal (*P* = 0.044) and 1.5 mm average to the fovea (*P* = 0.004), and it was relatively thicker than CNV scar at 3.0 mm average to the fovea (*P* = 0.017). ST, average relative and absolute PS height showed no significant difference between the subgroups of myopic CNV. The presence of scleral perforating vessels in the area of a CNV were found in 59 out of 75 (78.67%) eyes with myopic CNV. The rate of scleral perforating vessels found beneath or around myopic CNV showed no significant difference between the subgroups of myopic CNV (*P* = 0.713).

**Table 4:** The comparison of parameters on SS-OCT among subgroups of myopic CNV.

	**Active CNV (*n* = 39)**	**Scar/Fuch's spot (*n* = 20)**	**CNV-related macular atrophy (*n* = 16)**	***P***
Age, y	59.64 ± 11.90	53.40 ± 14.93	62.13 ± 5.80	0.221
Axial length, mm	29.09 ± 1.38	30.04 ± 1.56	29.82 ± 1.24	0.060
BCVA, logMAR	0.64 ± 0.38	0.45 ± 0.27	1.55 ± 0.78	<0.001^*^
CNV location (subfoveal/juxtafoveal)	31/8	7/13	16/0	<0.001^*^
CNV size, mm^2^	0.50 ± 0.55	0.28 ± 0.21	1.58 ± 0.36	0.002^*^
**ST**, **μm**				
Subfoveal	306.97 ± 93.04	274.75 ± 60.11	268.13 ± 76.61	0.452
1.5 mm average	246.72 ± 65.65	214.75 ± 53.46	225.86 ± 64.49	0.165
3.0 mm average	220.74 ± 51.88	203.68 ± 43.70	189.27 ± 59.40	0.102
**CT**, **μm (8 eyes were available in CNV-related macular atrophy group)**				
Subfoveal	19.23 ± 8.27	14.65 ± 9.48	12.38 ± 10.80	0.044^*^
1.5mm average	24.78 ± 9.33	19.16 ± 12.62	15.72 ± 12.15	0.004^*^
3.0mm average	25.43 ± 8.51	18.68 ± 13.10	22.59 ± 12.50	0.017^*^
Scleral perforating vessels (%)	76.92%	85.00%	75.00%	0.713
Average relative PS height, μm	433.52 ± 172.17	398.46 ± 162.02	453.30 ± 97.64	0.460
Average absolute PS height, μm	515.28 ± 132.38	472.06 ± 149.00	457.80 ± 90.42	0.350

*SS-OCT, swept-source optical coherence tomography; CNV, myopic choroidal neovascularization; BCVA, best-corrected visual acuity; logMAR, logarithm of minimum angle of resolution; ST, scleral thickness; CT, choroidal thickness; PS, posterior staphyloma*.

* *Significant difference*.

**Figure 4:**
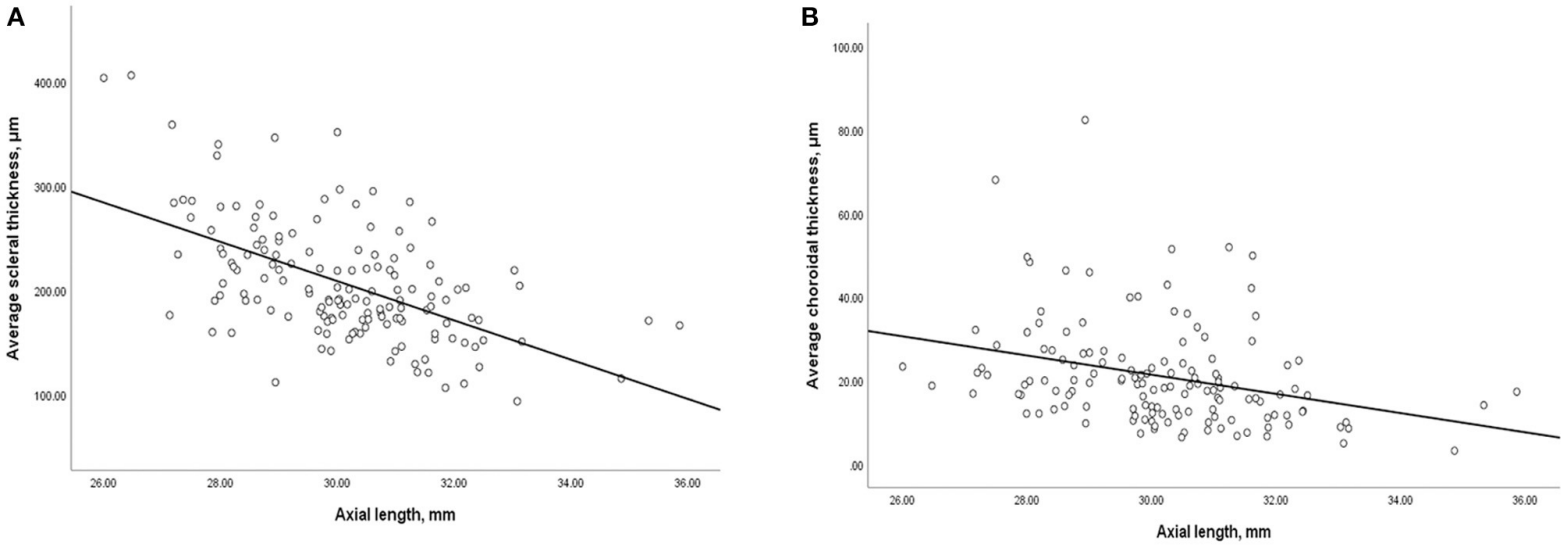
Scatterplot showing **(A)** negative correlation between average scleral thickness and axial length, **(B)** negative correlation between average choroidal thickness and axial length.

**Figure 5:**
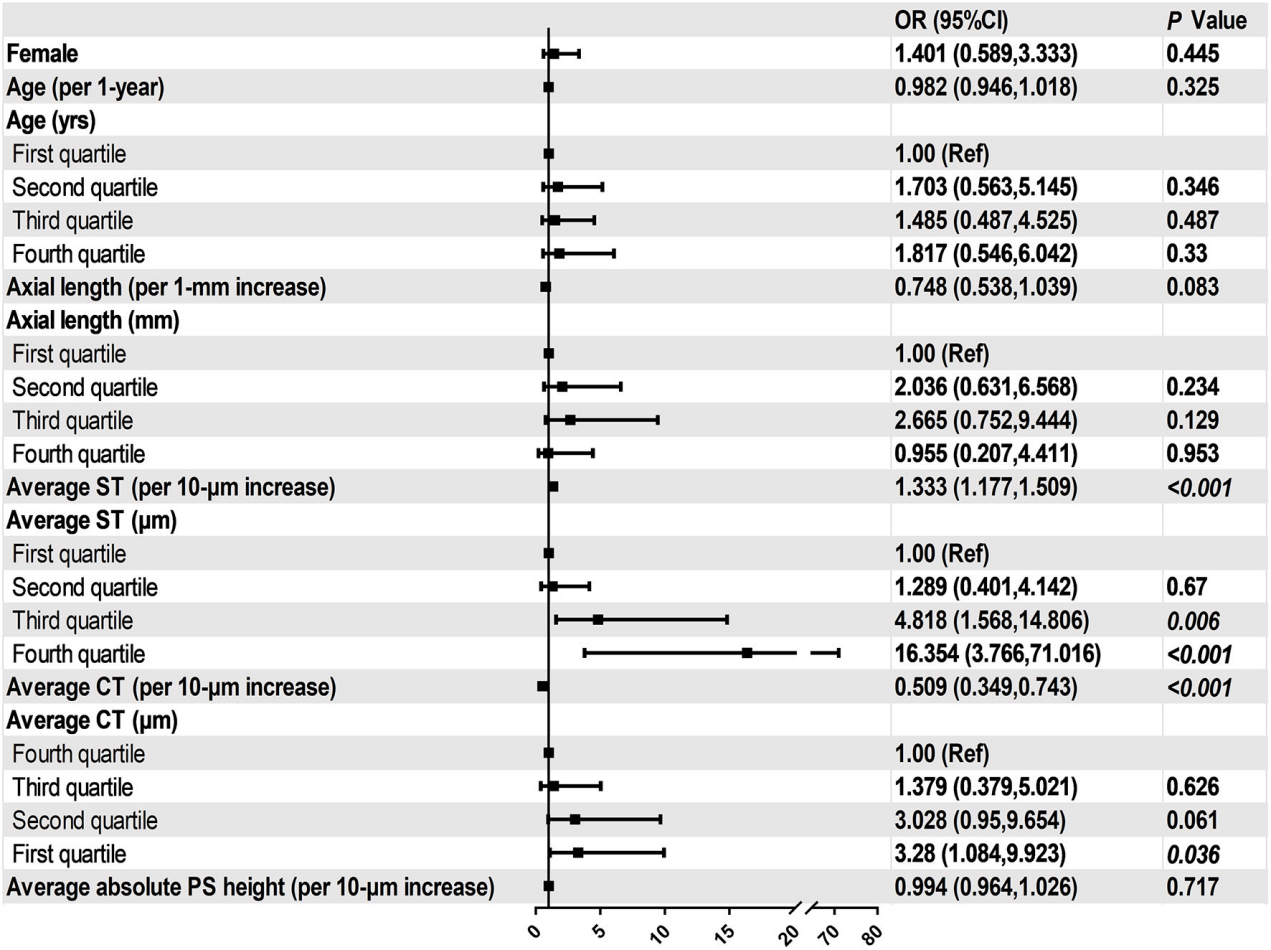
Associations between ocular parameters and the presence of myopic CNV in the multivariate adjusted models.

In all eyes without DSM, AL was inversely and significantly correlated with average ST (*r* = −0.5, *P* < 0.001) and average CT(*r* = −0.4, *P* < 0.001, Figure 4). Similarly, AL was negatively correlated with subfoveal ST (*r* = −0.5, *P* < 0.001) and subfoveal CT (*r* = −0.2, *P* = 0.007). We then performed binary logistic regression analysis to detect risk factors of myopic CNV (Figure 5). Eyes with thicker sclera (OR = 1.333, *P* < 0.001 per 10-μm increase) and thinner choroid (OR = 0.509, *P* = 0.010 per 10-μm increase) were more likely to have myopic CNV. After categorizing age, axial length, average ST and CT into quartiles, we found that eyes in the third (OR = 4.818, *P* = 0.006) and fourth quartile (OR = 16.354, *P* < 0.001) of average ST were significantly more likely to have myopic CNV. On the contrary, eyes in the first quartile (OR = 3.28, *P* = 0.036) of average CT were significantly more likely to have myopic CNV. Besides, larger average ST/CT (OR = 1.134, *P* = 0.002) and shorter axial length (OR = 0.56, *P* < 0.001) increased the possibility of myopic CNV occurrence with adjustment of age, gender and average absolute PS height.

## Discussion

To the best of our knowledge, this is the first study to recruit all phases of myopic CNV and compare the SS-OCT features between eyes with and without myopic CNV. The results of this study showed that the patients with myopic CNV had relatively shorter axial length and thicker sclera. After adjusting age, gender and axial length, thick sclera and thin choroid were associated with the presence of myopic CNV. In addition, scleral perforating vessels were detected in the area of myopic CNV in 78.67% of the subjects. These morphologic features are particularly important for screening myopic CNV in future investigations.

Previous studies have reported that the choroid thinning and choroidal filling delay was associated with the risk of developing myopic CNV (8, 9). However, in this study, we considered the parameters of ST and found that thicker sclera and thinner choroid may be biological indicators of CNV occurrence after adjusting age, gender and axial length. ST was reported to be significantly correlated with axial length (21), but it has rarely been discussed in the development of myopic CNV. In addition, Fang et al. (18) suggested that myopic CNV tended to develop in the eyes with an axial length of approximately 29 mm based on a long-term follow-up study. These all indicate that myopic CNV occurred during the progression of PM, but not in the late-stage of PM.

Scleral perforating vessels were reported to be associated with lacquer cracks and myopic CNV in previous studies (16, 22). In this study, approximately 80% of eyes with myopic CNV had scleral perforating vessels, this was consistent with the results of Ishida et al. (22) We tend to think that scleral perforating vessels were either concentrated forces of stretch from the sclera or the locus *minoris resistentiae* as previously reported (16) Due to the extremely thinned choroid caused by loss of large vessels, stroma and choriocapillaris, mechanical stress that is posed through scleral perforating vessels cannot be buffered and dispersed, and the force concentrated in a limited location which may cause the disruption of the RPE-BM-CC (retinal pigment epithelium-Bruch's membrane-choriocapillaris) complex, and myopic CNV may occur as an attempt to fix the mechanical break (23). Furthermore, the correlation between scleral thickness and amount of scleral perforating vessels requires sclera remodeling techniques and long-term follow-up.

The pathogenesis of myopic CNV is not fully understood but several hypotheses have been proposed, such as the mechanical theory, the heredodegenerative theory and the hemodynamic alteration in choroidal circulation (4, 23). This indicates that the presence of myopic CNV may be contributed by a combination of several factors. In this study, we described several characteristic features of myopic CNV on SS-OCT including a relatively thicker sclera and a thinner choroid with the presence of scleral perforating vessels. We especially filled the gap in the current understanding of scleral morphology in myopic CNV. Considering all the evidences above, we speculate that the unmatched decrease in scleral and choroidal thickness during the progression of pathologic myopia, with simultaneous mechanical effects caused by scleral perforating vessels may increase the risk of myopic CNV occurrence. Further studies recruiting newly onset myopic CNV patients are required to investigate whether the average ST/CT is a prognostic value to predict the occurrence of myopic CNV.

The correlation between staphyloma depth and the risk of myopic CNV is controversial. The nasal absolute staphyloma height was described to be associated with the myopic CNV occurrence in a small-scale study (8). However, we did not find any correlation between staphyloma height and myopic CNV occurrence. This is consistent with a previous study that myopic CNV usually occurs in the eyes with staphylomas of intermediary depth, and eyes with the deepest staphylomas had no myopic CNV indeed (24). These indicated that myopic CNV is a consequence of local pathological changes instead of the overall mechanical strength effect during the progressive elongation of the eyeball.

This study had several limitations. Firstly, this is a cross-sectional study, further long-term observational studies are required to detect whether myopic CNV develops above the scleral perforating vessels. Secondly, OCT and fundus photograph with a wide-field range of scanning may be more efficient to detect and measure PS in future analysis. Thirdly, reconstruction of choroid and scleral, as well as the corresponding myopic CNV lesions by artificial intelligence may be more comprehensive and intuitive for us to realize the morphologic features of myopic CNV. Fourthly, we did not include eyes with lacquer cracks in myopic CNV groups. Although lacquer cracks were risk factors for CNV, the progression of lacquer cracks to CNV was uncommon (18, 25). Further longitudinal studies and new classification system may be necessary for future investigations. Finally, SS-OCT acquired only radial scan images focusing on the macula. Certain characteristic morphologies may have been missed in the para- or extra-foveal regions.

In summary, we found that relatively thicker sclera and thinner choroid were biological indicators for myopic CNV on SS-OCT after adjusting age, gender, axial length. Additionally, scleral perforating vessels may be another pivotal factor associated with the presence of myopic CNV. The specific area above the scleral perforating vessels should be given special attention for early detection of myopic CNV. SS-OCT is a useful tool to identify the high-risk groups when screening large populations. Eyes with such morphologic features presented on SS-OCT may need closer follow-ups for monitoring myopic CNV.

## Data Availability Statement

The original contributions presented in the study are included in the article/supplementary materials, further inquiries can be directed to the corresponding author/s.

## Ethics Statement

This study was approved by the ethics committee of Shanghai General Hospital. Written informed consent was obtained from the individual(s) for the publication of any potentially identifiable images or data included in this article.

## Author Contributions

JX, YF, and XX designed this study. JX, QC, JY, HZ, and WW collected and measured data. JX and JH analyzed data. JX, QC, and YF wrote this article. All authors discussed the results and commented on the manuscript.

## Conflict of Interest

The authors declare that the research was conducted in the absence of any commercial or financial relationships that could be construed as a potential conflict of interest.
